# Design, synthesis and antimicrobial evaluation of novel 2-aryl-thiazolidin-4-one derivatives

**DOI:** 10.1186/2191-2858-1-4

**Published:** 2011-08-03

**Authors:** Davinder Prasad, Awanit Kumar, Praveen Kumar Shukla, Mahendra Nath

**Affiliations:** 1 Department of Chemistry, University of Delhi, Delhi 110 007, India; 2 Fermentation Technology Division, Central Drug Research Institute, Lucknow 226 001, India

**Keywords:** 2-arylthiazolidinones, antimicrobial activity, 1,2,4-triazole, imidazole, morpholine

## Abstract

Novel 2-arylthiazolidin-4-one derivatives (**8a-q **and **11**) have been synthesized in good-to-excellent yields (70-96%) by one-pot three-component condensation-cyclization reaction of aromatic or aliphatic primary amines, aromatic aldehydes, and thioglycolic acid in polypropylene glycol at 110°C temperature. The *in vitro *antimicrobial activity of the synthesized 2-arylthiazolidin-4-ones was investigated against a panel of six pathogenic fungal strains, a Gram-positive and three Gram-negative bacteria. Results revealed that the compounds (**8a-d**) bearing 3-(4-(1*H*-imidazolylmethyl)phenyl)-substituent displayed significant antibacterial efficacy specifically against *Klebsiella pneumoniae *(minimum inhibitory concentration 12.5 μg/mL). In addition, some of the synthesized compounds have also shown antimicotic activity against *Sporothrix schenckii*, *Trichophyton mentagrophytes*, and *Aspergillus fumigatus *at the concentration of 50 μg/mL.

**Graphical abstract:**

A series of novel 2-arylthiazolidin-4-one analogues was prepared and assessed for their *in vitro *antimicrobial efficacy. Some of the synthesized compounds displayed significant antibacterial efficacy against *Klebsiella pneumoniae *and selective antimycotic activity against *Trichophyton mentagrophytes*.

## Introduction

The increasing cases of microbial resistance pose a major concern to the scientific community and have become a threat for human life worldwide. Moreover, invasive microbial infections caused by multi-drug-resistant Gram-positive bacteria and microbes are difficult to diagnose and treat [1]. They are the major cause of morbidity and mortality especially in immunosuppressed and hospital-acquired patients [2]. To overcome these problems, the development of new and safe antimicrobial agents with better effectiveness is urgently required. To this end, one of the best ways to design new antimicrobial agents is to generate hybrid molecules by combining two bioactive heterocyclic moieties in a single molecular scaffold.

Among pharmacologically important heterocyclic compounds, 4-thiazolidinone derivatives have been known to possess a wide range of biological properties such as anticonvulsant [3], anti-HIV [4], antifungal [5], antibacterial agents [6], and COX-1 inhibitors [7]. In addition, the five- and six-membered heterocycles, such as imidazole, triazole, and morpholine are of great interest due to their presence in many pharmaceutical agents [8-10]. Owing to the biological significance of these two classes of compounds and in continuation of our ongoing study on antimicrobial agents [11], we planned to synthesize a combined molecular framework that involves these two different chromophores. Thus, a series of 2-arylthiazolidin-4-ones bearing imidazole, triazole, or morpholine moiety has been synthesized using one-pot three-component methodology [12] to investigate their antibacterial and antimycotic efficacy.

## Results and discussion

### Chemistry

**Scheme 1:**
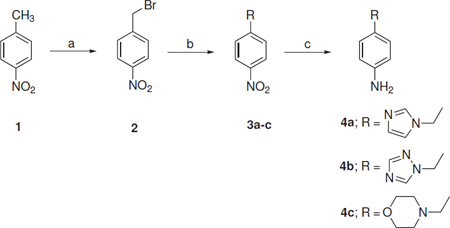
**Synthetic routes to 4-substituted phenylamines (4a-c)**. Reagents and conditions: (a) NBS, benzoyl peroxide, CCl_4_, reflux, 4-5 h; (b) DBU, imidazole or 1,2,4-triazole or morpholine, THF, room temperature, 20-24 h; (c) SnCl_2 _2H_2_O, conc. HCl, 50-60°C, 5-6 h.

**Scheme 2:**
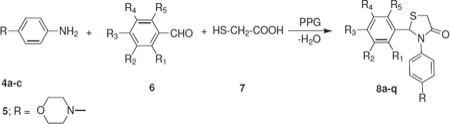
**Synthetic routes to 2-arylthiazolidin-4-one derivatives (8a-q)**.

**Scheme 3:**
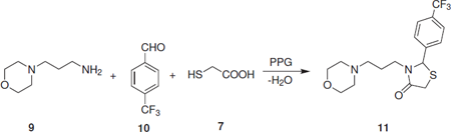
**Synthesis of 3-(3-morpholinopropyl)-2-(4-(trifluoromethyl)phenyl)thiazolidin-4-one (11)**.

The starting materials, 4-((1*H*-imidazol-1-yl)methyl)aniline (**4a**), 4-((1*H*-1,2,4-triazol-1-yl)methyl)aniline (**4b**), and 4-(morpholinomethyl)aniline (**4c**) were prepared from 4-nitro-toluene in three steps. The bromination of 4-nitrotoluene (**1**) with *N*-bromosuccinimide in the presence of catalytic amount of benzoyl peroxide was accomplished in carbon tetrachloride at reflux temperature. The resulting product, *p*-nitrobenzyl bromide (**2**), was then coupled with imidazole, 1,2,4-triazole, or morpholine according to the reported procedure [13], in the presence of DBU as a base in THF at ambient temperature to afford compounds (**3a-c**) which on subsequent reduction with SnCl_2 _in hydrochloric acid at 50-60°C yielded products (**4a-c; **Scheme Scheme 1). The physical and spectral data of compounds (**4a-c**) are in agreement with the reported data [14-16]. 4-Morpholinobenzenamine (**5**) and 3-morpholinopropan-1-amine (**9**) were purchased from Sigma-Aldrich and used without further purification. The synthetic routes to the target compounds are outlined in Schemes Scheme 2 and 3. For the synthesis of 2-arylthiazolidin-4-ones (**8a-q **and **11**), the condensation-cyclization reactions of various amines (**4a-c**, **5**, and **9**), aromatic aldehydes, and thioglycolic acid were performed at 110°C using polypropylene glycol (PPG) as a solvent medium. Initially, we attempted the synthesis of 3-(4-((1*H*-imidazol-1-yl)methyl)phenyl)-2-phenylthiazolidin-4-one (**8a**) by reacting 4-((1*H*-imidazol-1-yl)methyl)aniline (**4a**) with benzaldehyde and thioglycolic acid at 110°C in polyethylene glycol (PEG), as many organic transformations and multi-component reactions are reported in PEG, but surprisingly, no product formation was observed even after 24 h of the reaction. However, the reaction proceeds well in PPG under same reaction conditions and afforded the proffered product **8a **in 83% yield. The success of the reaction in PPG is possibly due to its immiscibility with water, which helps in the removal of a water molecule from the reaction mixture during the formation of 4-thiazolidinone ring. In addition, PPG is an eco-friendly solvent and associated with many advantages, such as low cost, less toxicity, efficient recyclability, easy work-up, and miscibility with a wide range of organic solvents.

The newly synthesized compounds were characterized by IR, ^1^H NMR, ^13^C NMR, ESI-MS, and elemental analysis. The formation of 3-(4-((1*H*-imidazol-1-yl)methyl)phenyl)-2-phenylthiazolidin-4-one (**8a**) was first indicated by IR. In IR spectrum, the compound **8a **exhibited a sharp band at 1686 cm^-1 ^because of CO stretching. The structure of compound **8a **was confirmed by NMR spectroscopy. In ^1^H NMR, the appearance of two doublets at δ 3.99 and 3.86 ppm due to geminal coupling between two hydrogens of S-*CH*_2_-CO and a sharp singlet at δ 6.08 ppm corresponding to N-*CH*-S indicated the presence of thiazolidinone ring. Similarly, three carbon signals at δ 33.30, 65.24, and 171.05 ppm in the ^13^C NMR of **8a **were assigned to S-*CH_2_*, N-*CH*-S, and *CO *groups, respectively. The mass spectral analysis produced further evidence for the formation of **8a **by showing [M]^+ ^ion peak at *m/z *335 for the molecular formula, C_19_H_17_N_3_OS. The general synthetic method and characterization data of compounds (**8a-q **and **11**) are described in the "Experimental section."

### Biological evaluation

The *in vitro *antibacterial activities of compounds (**8a-q **and **11**) and standard drugs (gentamycin and ampicillin) were carried out against a Gram-positive bacterial strain viz. *Staphylococcus aureus* (Sa) and three Gram-negative bacteria viz. *Escherichia coli* (Ec), *Klebsiella pneumoniae* (Kp) and *Pseudomonas aeruginosa* (Pa). The results of preliminary *in vitro *antibacterial testing are shown in Table 1. Out of 18 newly synthesized compounds, only 7 compounds (**8a-f **and **q**) were found to be active against the tested bacterial strains. 4-Thiazolidinones (**8a-f**) bearing 4-(1*H*-imidazolylmethyl)phenyl)-substituent at position 3 were found significantly active (minimum inhibitory concentration--MIC 12.5-50 μg/mL) against Gram-negative strain Kp. In addition, compounds (**8a-c**) have also shown moderate activity against Sa. In contrast, thiazolidinone **8q **with 3-(4-morpholinophenyl)-substituent exhibited activity (50 μg/mL) specifically against a Gram-negative bacterial strain Ec, which was found to be comparable with the standard drug ampicillin. Surprisingly, 4-thiazolidinone derivatives containing 4-(1,2,4-triazolylmethyl)phenyl- and 4-(morpholinomethyl)phenyl-functionalities at position 3 were found inactive against all the tested bacterial strains. These results imply that the nature of substituent at position 3 of thiazolidinone ring is responsible for antibacterial activity.

**Table 1:** *In vitro *antibacterial activities of compounds 8a-q and 11

Compound	*R*	*R*_1_	*R*_2_	*R*_3_	*R*_4_	*R*_5_	MIC (μg/mL)			
							
							Ec	Pa	Sa	Kp
**8a**	****	H	H	H	H	H	> 50	> 50	50	12.5
**8b**	****	H	H	CF_3_	H	H	> 50	> 50	50	12.5
**8c**	****	H	H	Br	H	H	> 50	> 50	50	12.5
**8d**	****	H	H	Cl	H	H	> 50	> 50	> 50	12.5
**8e**	****	H	H	CH_3_	H	H	> 50	> 50	> 50	25
**8f**	****	H	H	F	H	H	> 50	> 50	> 50	50
**8g**	****	CF_3_	H	H	H	H	> 50	> 50	> 50	> 50
**8h**	****	F	F	F	F	F	> 50	> 50	> 50	> 50
**8i**	****	H	H	Br	H	H	> 50	> 50	> 50	> 50
**8j**	****	H	H	F	H	H	> 50	> 50	> 50	> 50
**8k**		H	H	Br	H	H	> 50	> 50	> 50	> 50
**8l**	****	H	H	CF_3_	H	H	> 50	> 50	> 50	> 50
**8m**	****	H	H	Br	H	H	> 50	> 50	> 50	> 50
**8n**	****	H	H	Cl	H	H	> 50	> 50	> 50	> 50
**8o**	****	H	Br	H	H	H	> 50	> 50	> 50	> 50
**8p**	****	H	Cl	H	H	H	> 50	> 50	> 50	> 50
**8q**	****	H	F	H	H	H	50	> 50	> 50	> 50
**11**	-	-	-	-	-	-	> 50	> 50	> 50	> 50
Gentamycin	-	-	-	-	-	-	0.78	0.78	0.39	0.78
Ampicillin	-	-	-	-	-	-	50	50	0.19	0.39

Ec, *E. coli *(ATCC 9637); Pa, *P. aeruginosa *(ATCC BAA-427); Sa, *S. aureus *(ATCC 25923); Kp, *K. pneumonia *(ATCC 27736)

Furthermore, the *in vitro *antifungal activity of compounds (**8a-q **and **11**) was investigated along with standard drugs Ketoconazole and Fluconazole against a panel of six fungal strains, viz., *Candida albicans *(Ca), *Cryptococcus neoformans *(Cn), *Sporothrix schenckii *(Ss), *Trichophyton mentagrophytes *(Tm), *Aspergillus fumigatus *(Af), and *Candida parapsilosis *(Cp) and the results are presented in Table 2. Although, none of the compounds showed better efficacy than the standard drugs Ketoconazole and Fluconazole against the tested fungi, 12 of the title compounds (**8a**, **8g-q**) were found to be equipotent against Tm with MIC value of 50 μg/mL. The antifungal activity profile of these molecules seems to be dependent mainly on the substitution at position 3 of the thiazolidinone ring. Compounds **8g-i **containing 4-(1,2,4-triazolylmethyl)phenyl-moiety were found to be equipotent (MIC 50 μg/mL) against two fungal pathogens, Ss and Tm, whereas the compounds **8a **with 4-(1*H*-imidazolylmethyl)phenyl)-substituent and **8k-q **with 4-(morpholinomethyl)phenyl- or 4-morpholinophenyl-group displayed selective efficacy (MIC 50 μg/mL) against Tm. The remaining compounds (**8b-f**) having 4-(1*H*-imidazolylmethyl)phenyl)-group at position 3 of the thiazolidinone ring were found inactive against the screened fungi. Similarly, 3-(3-morpholinopropyl)-2-(4-(trifluoromethyl)phenyl)thiazolidin-4-one (**11**) did not show any activity against the tested microbial strains. These results indicate that the thiazolidinone ring does not have any influence on the activity profile of these molecules. However, the heteroaromatic moiety present at position 3 in the thiazolidinone ring seems to be responsible for the antimicrobial activity.

**Table 2:** *In vitro *antifungal activities of compounds 8a-q and 11

Compound	MIC (μg/mL)					
	
	Ca	Cn	Ss	Tm	Af	Cp
**8a**	> 50	> 50	> 50	50	> 50	> 50
**8b**	> 50	> 50	> 50	> 50	> 50	> 50
**8c**	> 50	> 50	> 50	> 50	> 50	> 50
**8d**	> 50	> 50	> 50	> 50	> 50	> 50
**8e**	> 50	> 50	> 50	> 50	> 50	> 50
**8f**	> 50	> 50	> 50	> 50	> 50	> 50
**8g**	> 50	> 50	50	50	> 50	> 50
**8h**	> 50	> 50	50	50	> 50	> 50
**8i**	> 50	> 50	50	50	> 50	> 50
**8j**	> 50	> 50	> 50	50	50	> 50
**8k**	> 50	> 50	> 50	50	> 50	> 50
**8l**	> 50	> 50	> 50	50	> 50	> 50
**8m**	> 50	> 50	> 50	50	> 50	> 50
**8n**	> 50	> 50	> 50	50	> 50	> 50
**8o**	> 50	> 50	> 50	50	> 50	> 50
**8p**	> 50	> 50	> 50	50	> 50	> 50
**8q**	> 50	> 50	> 50	50	> 50	> 50
**11**	> 50	> 50	> 50	> 50	> 50	> 50
Ketoconazole	0.002	0.001	0.031	4.0	2.0	0.031
Fluconazole	0.5	1.0	2.0	1.0	2.0	1.0

Ca, *C. albicans*; Cn, *C. neoformans*; Ss, *S. schenckii*; Tm, *T. mentagrophytes*; Af, *A. fumigatus*; Cp, *C. parapsilosis *(ATCC-22019).

## Conclusions

In summary, various 2-arylthiazolidin-4-ones (**8a-q**) and 3-(3-morpholinopropyl)-2-(4-(trifluoromethyl)phenyl)thiazolidin-4-one (**11**) have been synthesized via one-pot three-component methodology using PPG as a solvent medium and screened for their *in vitro *antimicrobial efficacy. It was observed that, instead of thiazolidinone ring, the heteroaromatic moiety at position 3 contributes to the antimicrobial activity. Compounds bearing imidazolylmethylphenyl substituent at position 3 of thiazolidinone ring showed significant antibacterial efficacy (MIC 12.5-50 μg/mL) against Gram-negative strain Kp, while compounds with the corresponding 1,2,4-triazole and morpholine substituents displayed comparable antimycotic activity (MIC 50 μg/mL) against Tm. Furthermore, these results may be useful for the designing of potent new antimicrobial agents.

## Experimental

### Chemistry

All the chemicals were purchased from Sigma-Aldrich and used without any further purification. Thin-layer chromatography was performed on precoated Merck silica gel 60 F254 plates, and spots were developed under UV light (254 nm) or in iodine chamber. All the compounds were purified by column chromatography using silica gel (60-120 mesh). The ^1^H NMR spectra were recorded on Bruker 300 or Jeol 400 MHz spectrometer. The IR spectra were obtained on a Perkin Elmer IR spectrometer, and peaks are given in reciprocal centimeter (cm^-1^). Mass spectra were recorded on Waters Micromass LCT ESI-MS spectrometer in positive ionization mode. Elemental analyses were determined on Elementar Analysensysteme GmbH VarioEL V3.00, and CHNS values were found within ± 0.4 of theoretical values for all the new compounds. The melting points were obtained by Perkin Elmer differential scanning calorimetry.

#### *General procedure for the synthesis of 8a-q *and *11*

A mixture of amine (1 mmol), aldehyde (2 mmol), and thioglycolic acid (3 mmol) in PPG ~2000 (2 mL) was heated at 110°C for 4-11 h. After completion of the reaction as indicated by TLC, the reaction mixture was diluted with hexane, and the precipitated product was filtered. In the case of oily products, the hexane layer was decanted, and sticky material was dissolved in ethyl acetate (20 mL). The solution was washed well with saturated NaHCO_3 _solution (15 mL × 3 times) followed by water (15 mL × 3 times). The organic layer was dried over anhydrous sodium sulfate and evaporated under reduced pressure to afford the crude compound. The product was purified by column chromatography on silica gel using 2-4% MeOH in benzene as eluent. In addition, the hexane layer was evaporated under reduced pressure to recover PPG which can be recycled.

#### 3-(4-((1H-imidazol-1-yl)methyl)phenyl)-2-phenylthiazolidin-4-one (8a)

Brown gummy oil; yield 83%. IR (CHCl_3_) ν: 1686 (C = O), 1513, 1425, 1356, 1273, 1217 1137, 1017, 957, 899, 863, 821, 755, 679 cm^-1^; ^1^H NMR (300 MHz, CDCl_3_) δ: 7.53 (s, 1 H, imidazole H), 7.28-7.26 (m, 4 H, ArH), 7.17 (d, *J *= 8.7 Hz, 2 H, ArH), 7.06 (s, 1 H, imidazole H), 7.03 (d, *J *= 8.4 Hz, 3 H, ArH), 6.83 (s, 1 H, imidazole H), 6.08 (s, 1 H, CH), 5.03 (s, 2 H, CH_2_-Ph) 3.99 (d, *J *= 15.9 Hz, 1 H, CH_2_), 3.86 (d, *J *= 15.9 Hz, 1 H, CH_2_) ppm; ^13^C NMR (100 MHz, CDCl_3_) δ: 171.05, 139.09, 137.20, 134.61, 129.29, 128.91, 128.86, 127.69, 126.71, 125.75, 119.25, 65.24, 50.02, 33.30 ppm; MS (ESI): *m/z *335 [M]^+^.

#### 3-(4-((1 H-imidazol-1-yl)methyl)phenyl)-2-(4-(trifluoromethyl)phenyl)thiazolidin-4-one (8b)

Yellow solid; m.p. 166°C; yield 86%. IR (CHCl_3_) ν: 1683 (C = O), 1515, 1380, 1326, 1232, 1166, 1123, 1067, 1017, 853, 753, 663 cm^-1^; ^1^H NMR (400 MHz, CDCl_3_) δ: 7.56 (d, *J *= 8.2 Hz, 2 H, ArH), 7.50 (s, 1 H, imidazole H), 7.40 (d, *J *= 8.2 Hz, 2 H, ArH), 7.18 (dd, *J*_1 _= 7.7 Hz, *J*_2 _= 1.83 Hz, 2 H, ArH), 7.07 (s, 1 H, imidazole H), 7.05 (d, *J *= 8.7 Hz, 2 H, ArH), 6.83 (s, 1 H, imidazole H), 6.14 (s, 1 H, CH), 5.04 (s, 2 H, CH_2_Ph), 3.98 (dd, *J*_1 _= 16.0 Hz, *J*_2 _= 1.3 Hz, 1 H, CH_2_), 3.87 (d, *J *= 15.5 Hz, 1 H, CH_2_) ppm; ^13^C NMR (100 MHz, CDCl_3_) δ: 170.91, 143.30, 137.04, 135.03, 129.52, 127.85, 127.06, 126.01, 125.97, 125.54, 64.39, 49.99, 33.21 ppm; MS (ESI): *m/z *404 [M + H]^+^; Anal. calcd for C_20_H_16_F_3_N_3_OS.1.1 H_2_O: C, 56.76; H, 4.33; N, 9.93; S, 7.58. Found: C, 56.63; H, 4.08; N, 9.55; S, 7.59.

#### 3-(4-((1 H-imidazol-1-yl)methyl)phenyl)-2-(4-bromophenyl)thiazolidin-4-one (8c)

Yellow solid; m.p. 180°C; yield 82%. IR (CHCl_3_) ν: 1683 (C = O), 1610, 1515, 1488, 1374, 1290, 1231, 1177, 1107, 1073, 1030, 1010, 906, 820, 752, 663 cm^-1^; ^1^H NMR (400 MHz, CDCl_3_) δ: 7.49 (s, 1 H, imidazole H), 7.42 (dd, *J*_1 _= 6.4 Hz, *J*_2 _= 1.8 Hz, 2 H, ArH), 7.16 (d, *J *= 1.3 Hz, 2 H, ArH), 7.14 (d, *J *= 1.3 Hz, 2 H, ArH), 7.07 (s, 1 H, imidazole H), 7.04 (d, *J *= 8.7 Hz, 2 H, ArH), 6.84 (s, 1 H, imidazole H), 6.05 (s, 1 H, CH), 5.05 (s, 2 H, CH_2_Ph), 3.96 (dd, *J*_1 _= 16.0 Hz, *J*_2 _= 1.3 Hz, 1 H, CH_2_), 3.86 (d, *J *= 16.0 Hz, 1 H, CH_2_) ppm; ^13^C NMR (100 MHz, CDCl_3_) δ: 171.01, 138.36, 137.27, 135.23, 132.30, 132.20, 128.71, 128.62, 127.91, 125.98, 125.89, 123.09, 64.83, 50.12, 33.45 ppm; MS (ESI): *m/z *413 [M]^+^; Anal. calcd for C_19_H_16_BrN_3_OS 0.2C_6_H_6 _0.8H_2_O: C, 54.60; H, 4.26; N, 9.46; S, 7.22. Found: C, 54.66; H, 4.12; N, 9.05; S, 7.59.

#### 3-(4-((1 H-imidazol-1-yl)methyl)phenyl)-2-(4-chlorophenyl)thiazolidin-4-one (8d)

Yellow solid; m.p.160°C; yield 78%. IR (CHCl_3_) ν: 1686 (C = O), 1508, 1376, 1290, 1231, 1088, 1030, 1014, 906, 839, 749, 691, 662, 615 cm^-1^; ^1^H NMR (400 MHz, CDCl_3_) δ: 7.51 (s, 1 H, imidazole H), 7.28-7.27 (m, 1 H, ArH),7.24 (dd, *J*_1 _= 9.1 Hz, *J*_2 _= 1.8 Hz, 2 H, ArH), 7.22-7.20 (m, 1 H, ArH), 7.15 (dd, *J*_1 _= 6.6 Hz, *J*_2 _= 2.2 Hz, 2 H, ArH), 7.07 (s, 1 H, imidazole H), 7.03 (d, *J *= 8.7 Hz, 2 H, ArH), 6.84 (s, 1 H, imidazole H), 6.07 (s, 1 H, CH), 5.04 (s, 2 H, CH_2_Ph), 3.96 (dd, *J*_1 _= 16.0 Hz, *J*_2 _= 1.3 Hz, 1 H, CH_2_), 3.86 (dd, *J*_1 _= 15.5 Hz, *J*_2 _= 0.9 Hz, 1 H, CH_2_) ppm; ^13^C NMR (100 MHz, CDCl_3_) δ: 170.90, 137.63, 137.32, 137.13, 135.02, 134.77, 129.63, 129.16, 128.27, 127.78, 125.83, 119.30, 64.58, 50.02, 33.33 ppm; MS (ESI): *m/z *369 [M]^+^; Anal. calcd for C_19_H_16_ClN_3_OS 0.1 H_2_O: C, 61.40; H, 4.39; N, 11.31; S, 8.63. Found: C, 61.66; H, 4.12; N, 11.05; S, 8.48.

#### 3-(4-((1 H-imidazol-1-yl)methyl)phenyl)-2-p-tolylthiazolidin-4-one (8e)

Brown gummy matter; yield 87%. IR (CHCl_3_) ν: 1684 (C = O), 1505, 1372, 1107, 1076, 906, 821, 751, 663 cm^-1^; ^1^H NMR (400 MHz, CDCl_3_) δ: 7.49 (s, 1 H, imidazole H), 7.17 (d, *J *= 6.4 Hz, 2 H, ArH),7.15 (d, *J *= 5.5 Hz, 2 H, ArH), 7.09 (d, *J *= 8.2 Hz, 2 H, ArH), 7.05 (d, *J *= 9.1 Hz, 2 H, ArH), 7.02 (s, 1 H, imidazole H), 6.83 (s, 1 H, imidazole H), 6.05 (s, 1 H, CH), 5.03 (s, 2 H, CH_2_Ph), 3.97 (d, *J *= 15.5 Hz, 1 H, CH_2_), 3.84 (d, *J *= 15.5 Hz, 1 H, CH_2_), 2.29 (s, 3H, CH_3_) ppm; MS (ESI): *m/z *350 [M + H]^+^.

#### 3-(4-((1 H-imidazol-1-yl)methyl)phenyl)-2-(4-fluorophenyl)thiazolidin-4-one (8f)

Yellow solid; m.p. 70°C; yield 74%. IR (CHCl_3_) ν: 1686 (C = O), 1604, 1510, 1378, 1280, 1227, 1157, 1107, 1076, 1030, 906, 847, 823, 788, 753, 692, 663 cm^-1^; ^1^H NMR (400 MHz, CDCl_3_) δ: 7.48 (s, 1 H, imidazole H), 7.28-7.25 (m, 2 H, ArH),7.14 (dd, *J*_1 _= 6.6 Hz, *J*_2 _= 1.8 Hz, 2 H, ArH), 7.07 (s, 1 H, imidazole H), 7.03 (d, *J *= 8.2 Hz, 2 H, ArH), 6.98 (dd, *J*_1 _= 6.4 Hz, *J*_2 _= 1.8 Hz, 2 H, ArH), 6.83 (t, *J *= 0.92 Hz, 1 H, imidazole H), 6.08 (s, 1 H, CH), 5.04 (s, 2 H, CH_2_Ph), 3.96 (dd, *J*_1 _= 15.8 Hz, *J*_2 _= 1.8 Hz, 1 H, CH_2_), 3.87 (d, *J *= 16.0 Hz, 1 H, CH_2_) ppm; MS (ESI): *m/z *354 [M + H]^+^; Anal. calcd for C_19_H_16_FN_3_OS 0.4H_2_O: C, 63.28; H, 4.70; N, 11.65; S, 8.89. Found: C, 63.46; H, 4.58; N, 11.50; S, 8.66.

#### 3-(4-((1 H-1,2,4-triazol-1-yl)methyl)phenyl)-2-(2-(trifluoromethyl)phenyl)thiazolidin-4-one (8g)

Yellow solid; m.p. 176°C; yield 88%. IR (CHCl_3_) ν: 1691 (C = O), 1509, 1341, 1314, 1273, 1217, 1166, 1117, 1060, 1039, 1017, 958, 768, 724, 679, 651 cm^-1^; ^1^H NMR (300 MHz, CDCl_3_) δ: 8.01 (s, 1 H, triazole H), 7.94 (s, 1 H, triazole H), 7.61 (d, *J *= 7.8 Hz, 1 H, ArH), 7.54 (m, 2 H, ArH), 7.40-7.36 (m, 1 H, ArH), 7.30 (d, *J *= 8.4 Hz, 2 H, ArH), 7.17 (d, *J *= 8.4 Hz, 2 H, ArH), 6.56 (s, 1 H, CH), 5.26 (s, 2 H, CH_2_-Ph), 3.99 (d, *J *= 15.9 Hz, 1 H, CH_2_), 3.86 (d, *J *= 15.9 Hz, 1 H, CH_2_) ppm; MS (ESI): *m/z *405 [M + H]^+^; Anal. calcd for C_19_H_15_F_3_N_4_OS 0.3H_2_O: C, 55.68; H, 3.84; N, 13.67; S, 7.82. Found: C, 55.46; H, 3.62; N, 13.55; S, 7.62.

#### 3-(4-((1 H-1,2,4-triazol-1-yl)methyl)phenyl)-2-(pentafluorophenyl)thiazolidin-4-one (8h)

White solid; m.p. 120°C; yield 70%. IR (CHCl_3_) ν: 1692 (C = O), 1560, 1541, 1506, 1420, 1389, 1339, 1273, 1223, 1138, 1116, 1017, 994, 962, 760, 732, 679, 621 cm^-1^; ^1^H NMR (300 MHz, CDCl_3_) δ: 8.08 (s, 1 H, triazole H), 7.97 (s, 1 H, triazole H), 7.29 (d, *J *= 8.4 Hz, 2 H, ArH), 7.23 (d, *J *= 8.4 Hz, 2 H, ArH), 6.55 (s, 1 H, CH), 5.31 (s, 2 H, CH_2_-Ph), 4.09 (d, *J *= 15.6 Hz, 1 H, CH_2_), 3.87 (d, *J *= 15.6 Hz, 1 H, CH_2_) ppm; MS (ESI): *m/z *427 [M + H]^+^; Anal. calcd for C_18_H_11_F_5_N_4_OS.: C, 50.71; H, 2.60; N, 13.14; S, 7.52. Found: C, 50.61; H, 2.52; N, 13.45; S, 7.45.

#### 3-(4-((1 H-1,2,4-triazol-1-yl)methyl)phenyl)-2-(4-bromophenyl)thiazolidin-4-one (8i)

Yellow solid; m.p. 178°C; Yield 76%. IR (CHCl_3_) ν: 1687 (C = O), 1514, 1489, 1376, 1272, 1217, 1137, 1072, 1011, 958, 842, 751, 679 cm^-1^; ^1^H NMR (300 MHz, CDCl_3_) δ: 8.04 (s, 1 H, triazole H), 7.95 (s, 1 H, triazole H), 7.42 (d, *J *= 7.8 Hz, 2 H, ArH), 7.17 (m, 6H, ArH), 6.06 (s, 1 H, CH), 5.27 (s, 2 H, CH_2_-Ph), 3.96 (d, *J *= 15.0 Hz, 1 H, CH_2_), 3.85 (d, *J *= 15.9 Hz, 1 H, CH_2_) ppm; MS (ESI): *m/z *415 [M + H]^+^; Anal. calcd for C_18_H_15_BrN_4_OS 0.35H_2_O: C, 51.28; H, 3.75; N, 13.29; S, 7.61. Found: C, 51.36; H, 3.96; N, 12.98; S, 7.69.

#### 3-(4-((1 H-1,2,4-triazol-1-yl)methyl)phenyl)-2-(4-fluorophenyl)thiazolidin-4-one (8j)

Yellow solid; m.p. 130°C; yield 78%. IR (CHCl_3_) ν: 1686 (C = O), 1605, 1510, 1376, 1273, 1225, 1158, 1138, 1016, 958, 848, 789, 755, 679 cm^-1^; ^1^H NMR (300 MHz, CDCl_3_) δ: 8.02 (s, 1 H, triazole H), 7.95 (s, 1 H, triazole H), 7.27-7.24 (m, 2 H, ArH), 7.16 (m, 4 H, ArH), 6.97 (t, *J *= 8.4 Hz, 2 H, ArH), 6.09 (s, 1 H, CH), 5.26 (s, 2 H, CH_2_-Ph), 3.96 (d, *J *= 15.9 Hz, 1 H, CH_2_), 3.86 (d, *J *= 15.9 Hz, 1 H, CH_2_) ppm; MS (ESI): *m/z *355 [M + H]^+^; Anal. calcd for C_18_H_15_FN_4_OS 0.15 H_2_O: C, 60.54; H, 4.32; N, 15.69; S, 8.98. Found: C, 60.78; H, 4.02; N, 15.45; S, 8.85.

#### 2-(4-Bromophenyl)-3-(4-(morpholinomethyl)phenyl)thiazolidin-4-one (8k)

Gummy brown solid; yield 82%. IR (CHCl_3_) ν: 1693 (C = O), 1679, 1609, 1512, 1487, 1454, 1379, 1290, 1264, 1217, 1177, 1115, 1071, 1035, 1008, 914, 865, 838, 797, 754, 664, 644, 624 cm^-1^; ^1^H NMR (400 MHz, CDCl_3_) δ: 7.41 (dd, *J*_1 _= 6.6 Hz, *J*_2 _= 1.6 Hz, 2 H, ArH), 7.25 (d, *J *= 8.4 Hz, 2 H, ArH), 7.16 (dd, *J*_1 _= 6.7 Hz, *J*_2 _= 1.6 Hz, 2 H, ArH), 7.09 (d, *J *= 8.2 Hz, 2 H, ArH), 6.04 (s, 1 H, CH), 3.96 (dd, *J*_1 _= 15.8 Hz, *J*_2 _= 1.3 Hz, 1 H, CH_2_), 3.86 (d, *J *= 15.8 Hz, 1 H, CH_2_), 3.67 (t, *J *= 4.5 Hz, 4 H, morpholine H), 3.40 (s, 2 H, CH_2_-Ph), 2.38 (t, *J *= 4.3 Hz, 4 H, morpholine H) ppm; ^13^C NMR (100 MHz, CDCl_3_) δ: 170.83, 138.52, 136.71, 136.06, 131.97, 129.76, 128.54, 125.28, 122.75, 66.76, 64.82, 62.53, 53.43, 33.30 ppm; MS (ESI): *m/z *433 [M + H]^+^.

#### 3-(4-Morpholinophenyl)-2-(4-(trifluoromethyl)phenyl)thiazolidin-4-one (8l)

Dark brown gummy solid; m.p. 110°C; yield 94%. IR (CHCl_3_) ν: 1682 (C = O), 1609, 1516, 1450, 1421, 1379, 1325, 1263, 1165, 1120, 1067, 1017, 930, 851, 825, 803, 752, 659 cm^-1^; ^1^H NMR (400 MHz, CDCl_3_) δ: 7.55 (d, *J *= 8.2 Hz, 2 H, ArH), 7.41 (d, *J *= 8.2 Hz, 2 H, ArH), 7.02 (dd, *J*_1 _= 6.8 Hz, *J*_2 _= 2.0 Hz, 2 H, ArH), 6.78 (dd, *J*_1 _= 6.8 Hz, *J*_2 _= 2.0 Hz, 2 H, ArH), 6.04 (s, 1 H, CH), 3.99 (dd, *J*_1 _= 15.8 Hz, *J*_2 _= 1.6 Hz, 1 H, CH_2_), 3.88 (d, *J *= 15.8 Hz, 1 H, CH_2_), 3.79 (t, *J *= 4.8 Hz, 4 H, morpholine H), 3.09 (t, *J *= 5.0 Hz, 4 H, morpholine H) ppm; MS (ESI): *m/z *409 [M + H]^+^.

#### 2-(4-Bromophenyl)-3-(4-morpholinophenyl)thiazolidin-4-one (8m)

Bright yellow solid; m.p. 144°C; yield 85%. IR (CHCl_3_) ν: 1683 (C = O), 1608, 1516, 1488, 1449, 1408, 1378, 1334, 1292, 1263, 1234, 1178, 1121, 1071, 1052, 1009, 930, 824, 798, 757, 665, 648 cm^-1^; ^1^H NMR (400 MHz, CDCl_3_) δ: 7.41 (dd, *J*_1 _= 6.5 Hz, *J*_2 _= 1.8 Hz, 2 H, ArH), 7.17 (dd, *J*_1 _= 6.5 Hz, *J*_2 _= 1.8 Hz, 2 H, ArH), 6.98 (dd, *J*_1 _= 6.8 Hz, *J*_2 _= 2.0 Hz, 2 H, ArH), 6.78 (dd, *J*_1 _= 6.8 Hz, *J*_2 _= 2.0 Hz, 2 H, ArH), 5.95 (d, *J *= 1.3 Hz, 1 H, CH), 3.96 (dd, *J*_1 _= 15.8 Hz, *J*_2 _= 1.8 Hz, 1 H, CH_2_), 3.86 (d, *J *= 15.5 Hz, 1 H, CH_2_), 3.80 (t, *J *= 4.8 Hz, 4 H, morpholine H), 3.09 (t, *J *= 4.8 Hz, 4 H, morpholine H) ppm; ^13^C NMR (100 MHz, CDCl_3_) δ: 170.82, 149.99, 138.67, 131.86, 128.78, 128.62, 126.81, 122.70, 115.63, 66.61, 65.05, 48.60, 33.20 ppm; MS (ESI): *m/z *419 [M + H]^+^. Anal. calcd for C_19_H_19_BrN_2_O_2_S 0.15C_6_H_6_: C, 55.45; H, 4.65; N, 6.50; S, 7.44. Found: C, 55.45; H, 4.45; N, 6.37; S, 7.39.

#### 2-(4-Chlorophenyl)-3-(4-morpholinophenyl)thiazolidin-4-one (8n)

Yellow solid; m.p. 132°C; Yield 78%. IR (CHCl_3_) ν: 1683 (C = O), 1607, 1515, 1491, 1449, 1378, 1334, 1262, 1234, 1177, 1120, 1088, 1051, 1013, 930, 825, 756, 653 cm^-1^; ^1^H NMR (400 MHz, CDCl_3_) δ: 7.27-7.22 (m, 4 H, ArH), 6.98 (dd, *J*_1 _= 6.8 Hz, *J*_2 _= 2.3 Hz, 2 H, ArH), 6.78 (dd, *J*_1 _= 6.8 Hz, *J*_2 _= 2.0 Hz, 2 H, ArH), 5.96 (d, *J *= 1.6 Hz, 1 H, CH), 3.96 (dd, *J*_1 _= 15.7 Hz, *J*_2 _= 1.8 Hz, 1 H, CH_2_), 3.86 (d, *J *= 15.8 Hz, 1 H, CH_2_), 3.80 (t, *J *= 4.8 Hz, 4 H, morpholine H), 3.09 (t, *J *= 3.4 Hz, 4 H, morpholine H) ppm; ^13^C NMR (100 MHz, CDCl_3_) δ: 170.84, 150.02, 138.14, 134.52, 128.92, 128.65, 128.52, 126.86, 115.64, 66.62, 65.03, 48.63, 33.22 ppm; MS (ESI): *m/z *375 [M + H]^+^; Anal. calcd for C_19_H_19_ClN_2_O_2_S 0.15C_6_H_6_: C, 61.82; H, 5.19; N, 7.25; S, 8.29. Found: C, 61.73; H, 5.45; N, 7.24; S, 8.32.

#### 2-(3-Bromophenyl)-3-(4-morpholinophenyl)thiazolidin-4-one (8o)

Brown solid; m.p. 106°C; yield 91%. IR (CHCl_3_) ν: 1685 (C = O), 1608, 1593, 1573, 1516, 1474, 1449, 1431, 1378, 1330, 1262, 1234, 1179, 1121, 1070, 1052, 930, 902, 826, 796, 756, 686, 666 cm^-1^; ^1^H NMR (400 MHz, CDCl_3_) δ: 7.45-7.36 (m, 1 H, ArH), 7.36 (s, 1 H, ArH), 7.22-7.14 (m, 2 H, ArH), 7.01 (dd, *J*_1 _= 6.7 Hz, *J*_2 _= 2.0 Hz, 2 H, ArH), 6.79 (dd, *J*_1 _= 6.8 Hz, *J*_2 _= 2.3 Hz, 2 H, ArH), 5.92 (d, *J *= 1.6 Hz, 1 H, CH), 3.98 (dd, *J*_1 _= 15.7 Hz, *J*_2 _= 1.8 Hz, 1 H, CH_2_), 3.86 (d, *J *= 15.8 Hz, 1 H, CH_2_), 3.80 (t, *J *= 4.8 Hz, 4 H, morpholine H), 3.10 (t, *J *= 5.0 Hz, 4 H, morpholine H) ppm; MS (ESI): *m/z *419 [M + H]^+^; Anal. calcd for C_19_H_19_BrN_2_O_2_S: C, 54.42; H, 4.57; N, 6.68; S, 7.65. Found: C, 54.78; H, 4.33; N, 6.45; S, 7.85.

#### 2-(3-Chlorophenyl)-3-(4-morpholinophenyl)thiazolidin-4-one (8p)

Yellow solid; m.p. 122°C; yield 88%. IR (CHCl_3_) ν: 1686 (C = O), 1608, 1577, 1516, 1477, 1449, 1378, 1331, 1262, 1234, 1121, 1077, 1052, 930, 894, 860, 826, 798, 757, 685, 657, 615 cm^-1^; ^1^H NMR (400 MHz, CDCl_3_) δ: 7.36-7.29 (m, 1 H, ArH), 7.23-7.22 (m, 2 H, ArH), 7.17-7.16 (m, 1 H, ArH), 7.01 (dd, *J*_1 _= 6.8 Hz, *J*_2 _= 2.0 Hz, 2 H, ArH), 6.79 (dd, *J*_1 _= 7.0 Hz, *J*_2 _= 2.2 Hz, 2 H, ArH), 5.93 (d, *J *= 1.6 Hz, 1 H, CH), 3.98 (dd, *J*_1 _= 15.8 Hz, *J*_2 _= 1.8 Hz, 1 H, CH_2_), 3.86 (d, *J *= 15.8 Hz, 1 H, CH_2_), 3.80 (t, *J *= 4.8 Hz, 4 H, morpholine H), 3.09 (t, *J *= 4.8 Hz, 4 H, morpholine H) ppm; ^13^C NMR (100 MHz, CDCl_3_) δ: 170.85, 150.05, 141.94, 134.57, 130.03, 128.96, 128.68, 127.13, 126.79, 125.15, 115.69, 66.63, 65.01, 48.66, 33.14 ppm; MS (ESI): *m/z *375 [M + H]^+^; Anal. calcd for C_19_H_19_ClN_2_O_2_S 1.2 H_2_O: C, 57.55; H, 5.44; N, 7.07; S, 8.09. Found: C, 57.49; H, 5.34; N, 7.34; S, 8.19.

#### 2-(3-Fluorophenyl)-3-(4-morpholinophenyl)thiazolidin-4-one (8q)

Light yellow solid; m.p. 138°C; yield 95%. IR (CHCl_3_) ν: 1685 (C = O), 1609, 1593, 1516, 1488, 1450, 1379, 1333, 1305, 1263, 1234, 1177, 1143, 1121, 1072, 1052, 931, 902, 873, 826, 803, 754, 686, 665, 619 cm^-1^; ^1^H NMR (400 MHz, CDCl_3_) δ: 7.26-7.22 (m, 1 H, ArH), 7.07-6.89 (m, 5H, ArH), 6.79 (dd, *J*_1 _= 6.8 Hz, *J*_2 _= 2.2 Hz, 2 H, ArH), 5.96 (d, *J *= 1.4 Hz, 1 H, CH), 3.98 (dd, *J*_1 _= 15.7 Hz, *J*_2 _= 1.6 Hz, 1 H, CH_2_), 3.86 (dd, *J*_1 _= 15.8 Hz, *J*_2 _= 0.4 Hz, 1 H, CH_2_), 3.80 (t, *J *= 4.8 Hz, 4 H, morpholine H), 3.09 (t, *J *= 4.8 Hz, 4 H, morpholine H) ppm; ^13^C NMR (100 MHz, CDCl_3_) δ: 170.83, 150.00, 130.36, 130.27, 128.71, 126.75, 122.64, 115.92, 115.64, 114.08, 113.85, 66.62, 65.00, 48.63, 33.12 ppm; MS (ESI): *m/z *359 [M + H]^+^; Anal. calcd for C_19_H_19_FN_2_O_2_S: C, 63.67; H, 5.34; N, 7.82; S, 8.95. Found: C, 63.45; H, 5.45; N, 7.47; S, 8.74.

#### 3-(3-Morpholinopropyl)-2-(4-(trifluoromethyl)phenyl)thiazolidin-4-one (11)

Brown gummy solid; yield 96%. IR (CHCl_3_) ν: 1681 (C = O), 1619, 1447, 1410, 1325, 1273, 1209, 1165, 1117, 1067, 1017, 1034, 955, 917, 862, 813, 775, 750, 667 cm^-1^; ^1^H NMR (400 MHz, CDCl_3_) δ: 7.64 (d, *J *= 8.2 Hz, 2 H, ArH), 7.41 (d, *J *= 8.0 Hz, 2 H, ArH), 5.70 (d, *J *= 1.8 Hz, 1 H, CH), 3.80 (dd, *J*_1 _= 15.4 Hz, *J*_2 _= 1.8 Hz, 1 H, CH_2_), 3.73-3.66 (m, 2 H), 3.64 (t, *J *= 4.5 Hz, 4 H, morpholine H), 2.76-2.69 (m, 1 H, aliphatic CH_2_), 2.33 (t, *J *= 4.5 Hz, 4 H, morpholine H), 2.29-2.20 (m, 2 H, aliphatic CH_2_), 1.72-1.56 (m, 2 H, aliphatic CH_2_) ppm; ^13^C NMR (100 MHz, CDCl_3_) δ: 171.19, 143.76, 127.23, 126.09, 126.05, 66.64, 62.86, 55.57, 53.34, 41.28, 32.68, 23.43 ppm; MS (ESI): *m/z *375 [M + H]^+^.

#### In vitro antibacterial assay

The antibacterial activities of all the prepared compounds were evaluated against a Gram-positive bacterial strain viz, Kp and Sa, and a Gram-positive bacteria, viz., Sa aand three Gram-negative bacteria viz. Ec, Kp and Pa by the NCCLS Method using Mueller Hinton broth in 96-well tissue culture plates. The bacterial strains were grown on nutrient agar at 37°C. After 24 h of incubation, bacterial cells were suspended in normal saline containing Tween 20 at 0.05% concentration of approximately 1.0-2.0 × 10^7 ^cells/mL by matching with McFarland standards. Proper growth control, drug control, and the negative control were adjusted on to the plate. Compounds were dissolved in dimethyl sulfoxide at a concentration of 1 mg/mL, and 20 mL of this solution was added to each well of 96-well tissue culture plate having 180 mL of Mueller Hinton broth. The solution was then serially diluted to afford twofold serial dilutions of the test compounds in the subsequent wells. Then, McFarland-matched bacterial suspension (100 mL) was diluted with 10 mL of media. The diluted bacterial suspension (100 mL) was added to each well and then kept for incubation. The maximum concentration of compounds tested was 50 mg/mL. Microtitre plates were incubated at 35°C in a moist, dark chamber. MICs were recorded spectrophotometrically after 24 h incubation. Gentamycin and ampicillin were used as reference anti-bacterial agents.

#### In vitro antifungal assay

The *in vitro *antifungal activity of 4-thiazolidinone derivatives (**8a-q **and **11**) was investigated against six pathogenic fungi, viz., Ca, Cn, Ss, Tm, Af, and Cp by broth microdilution technique as per guidelines of the National Committee for Clinical Laboratory Standards [17] using RPMI Medium 1640 buffered with MOPS [3-(*N*-morpholino) propanesulphonic acid] in microtitre plates. The starting inoculums of test culture were 1-5 × 10^3 ^cfu/mL. Microtiter plates were incubated at 35°C. Minimal inhibitory concentrations were determined by spectrophotometric method at 492 nm after 24-48 h (yeasts) and 72-96 h (mycelial fungi) incubations. Ketoconazole and fluconazole were used as reference fungicides.

## Abbreviations

MIC: minimum inhibitory concentration; PEG: polyethylene glycol; PPG: polypropylene glycol.

## Competing interests

The authors declare that they have no competing interests.

## References

[B1] Goossens H European status of resistance in nosocomial infections Chemotherapy 2005 51 177 181 10.1159/000086919 16006764

[B2] Mathew BP Nath M Recent approaches to antifungal therapy for invasive mycoses ChemMedChem 2009 4 310 323 10.1002/cmdc.200800353 19170067

[B3] Dwivedi C Gupta TK Parmar SS Substituted thiazolidones as anticonvulsants J Med Chem 1972 15 553 554 (b) Parmar SS, Dwivedi C, Chaudhari A, Gupta TK (1972) Substituted thiazolidones and their selective inhibition of nicotinamide-adenine dinucleotide dependent oxidations. J Med Chem 15:99-101 10.1021/jm00275a031 5035284

[B4] Rawal RK Tripathi R Katti SB Pannecouque C Clercq ED Synthesis and evaluation of 2-(2,6-dihalophenyl)-3-pyrimidinyl-1,3-thiazolidin-4-one analogues as anti-HIV-1 agents Bioorg Med Chem 2007 15 3134 3142 (b) Rawal RK, Tripathi R, Katti SB, Pannecouque C, Clercq ED (2008) Design and synthesis of 2-(2,6-dibromophenyl)-3-heteroaryl-1,3-thiazolidin-4-ones as anti-HIV agents. Eur J Med Chem 43:2800-2806 10.1016/j.bmc.2007.02.044 17349793

[B5] Omar K Geronikaki A Zoumpoulakis P Camoutsis C Sokovic M Ciric A Glamoclija J Novel 4-thiazolidinone derivatives as potential antifungal and antibacterial drugs Bioorg Med Chem 2010 18 426 432 10.1016/j.bmc.2009.10.041 19914077

[B6] Vicini P Geronikaki A Anastasia K Incerti M Zani F Synthesis and antimicrobial activity of novel 2-thiazolylimino-5-arylidene-4-thiazolidinones Bioorg Med Chem 2006 14 3859 3864 10.1016/j.bmc.2006.01.043 16488614

[B7] Vigorita MG Ottana R Monforte F Maccari R Monforte MT Trovato A Taviano MF Miceli N Luca GD Alcaro S Ortuso F Chiral 3,3'-(1,2-ethanediyl)-bis[2-(3,4-dimethoxyphenyl)-4-thiazolidin-ones] with anti-inflammatory activity. Part 11: evaluation of COX-2 selectivity and modelling Bioorg Med Chem 2003 11 999 1006 (b) Previtera T, Vigorita MG, Basile M, Fenech G, Trovato A, Occhiuto F, Monforte MT, Barbera R (1990) 3,3'-Di(1,3-thiazolidine-4-one) system. V. Synthesis and pharmacological properties of 3,3'(1,2-ethanediyl)bis-(2-heteroaryl-1,3-thiazolidine-4-one) derivatives. Eur J Med Chem 25:569-579 10.1016/S0968-0896(02)00518-7 12614885

[B8] Letnicer D Organic chemistry of drug synthesis 2008 7 Wiley, New York

[B9] Al-Masoudi IA Al-Soud YA Al-Salihi NJ Al-Masoudi NA 1,2,4-Triazoles: synthetic approaches and pharmacological importance Chem Heterocycl Comp 2006 42 1377 1403 10.1007/s10593-006-0255-3

[B10] Saag MS Dismukes WS Azole antifungal agents: emphasis on new triazoles Antimicrob Agents Chemother 1988 32 1 8 2831809 10.1128/aac.32.1.1 PMC172087

[B11] Mathew BP Kumar A Sharma S Shukla PK Nath M An eco-friendly synthesis and antimicrobial activities of dihydro-2*H*-benzo- and naphtho-1,3-oxazine derivatives Eur J Med Chem 2010 45 1502 1507 10.1016/j.ejmech.2009.12.058 20116901

[B12] Prasad D Nath M Three-component domino reaction in PPG: an easy access to 4-thiazolidinone derivatives J Heterocycl Chem 2011

[B13] Bulger PL Cottrell IF Cowden CJ Davies AJ Dolling UH An investigation into the alkylation of 1,2,4-triazole Tetrahedron Lett 2000 41 1297 1301 10.1016/S0040-4039(99)02272-8

[B14] Roumen L Peeters JW Emmen JMA Beugels IPE Custers EMG Gooyer MD Plate R Pieterse K Hilbers PAJ Smits JFM Vekemans JAJ Leysen D Ottenheijm HCJ Janssen HM Hermans JJR Synthesis, biological evaluation, and molecular modeling of 1-benzyl-1*H*-imidazoles as selective inhibitors of aldosterone synthase (CYP11B2) J Med Chem 2010 53 1712 1725 10.1021/jm901356d 20121113

[B15] Street LJ Baker R Davey WB Guiblin AR Jelley RA Reeve AJ Routledge H Sternfeld F Watt AP Beer MS Middlemiss DN Noble AJ Stanton JA Scholey K Hargreaves RJ Sohal B Graham MI Matassat VG Synthesis and serotonergic activity of *N, N*-dimethyl-2-[5-(1,2,4-triazol-1-ylmethyl)-*1 H*-indol-3-yl]ethylamine and analogs: potent agonists for 5-HT_1D _receptors J Med Chem 1995 38 1799 1810 10.1021/jm00010a025 7752204

[B16] Dorow RL Herrinton PM Hohler RA Maloney MT Mauragis MA McGhee WE Moeslein JA Strohbach JW Veley MF Development of an efficient synthesis of the pyrrolquinolone PHA-529311 Org Process Res Dev 2006 10 493 499 10.1021/op050251y

[B17] National Committee for Clinical Laboratory Standards Reference method for broth dilution antifungal susceptibility testing of yeasts 1997 Approved Standard, NCCLS document M27-A: Wayne, PA, USA

